# A Joint Active Damping Strategy Based on *LCL*-Type Grid-Connected Inverters for Grid Current Feedback and PCC Voltage Unit Feedforward

**DOI:** 10.3390/s24186029

**Published:** 2024-09-18

**Authors:** Shanwen Ke, Bo Liang

**Affiliations:** School of Automation, Northwestern Polytechnical University, Xi’an 710072, China; kswkch@mail.nwpu.edu.cn

**Keywords:** active damping, grid current feedback (GCF), joint active damping, robustness, grid impedance

## Abstract

The negative high-pass filter feedback of the grid current (NFGCF) can offer active damping for the *LCL*-type grid-connected inverter. Due to the control delay in digital control systems, this damping can cause the system to exhibit non-minimum phase behavior within specific frequency ranges. This study proposes a joint active damping approach that combines grid current feedback and the point of common coupling (PCC) voltage unit feedforward. The proposed method introduces a dynamic damping region that varies with grid impedance. By developing suitable damping loop control parameters, this region can span the entire frequency range, even exceeding the Nyquist frequency *f*_s_/2. The research results demonstrate that the proposed approach enhances robustness against variations in grid impedance and eliminates non-minimum phase behavior. Simulation and experimental outcomes validate the effectiveness of this joint active damping method.

## 1. Introduction

Efficiently using renewable energy requires implementing distributed generation systems powered by renewable energy sources. These systems convert direct current to alternating current via grid-connected inverters, which is then fed into the electrical grid. The safe and stable operation of these grid-connected inverters is crucial for the energy conversion interface between new energy generation systems and traditional power systems [1,2,3]. In real applications, low-voltage power grids frequently exhibit weak grid characteristics, which challenge the operation of widely used *LCL*-type three-phase grid-connected inverters [4,5,6,7,8,9]. The impedance variation of the power grid can alter the system’s resonant frequency, leading to the failure of conventional damping methods and causing system instability [10,11,12,13,14].

Active damping strategies are commonly employed to decrease resonance in systems, with state variable feedback being the most commonly used method [15,16,17,18,19]. Capacitor-current and grid current feedback are two typical state-variable feedback damping methods used for system resonance suppression. Both methods are equivalent to paralleling a virtual resistor and a virtual reactance at both ends of the filtering capacitor. However, due to a fixed digital control delay, the virtual resistor exhibits negative values within certain frequency ranges [20,21,22], resulting in the emergence of a pair of open-loop right-half-plane (RHP) poles and the manifestation of non-minimum phase behavior in the system [23,24,25]. In order to achieve stable operation in this situation, the system must meet stricter requirements for amplitude–frequency characteristics. [26]. The virtual resistance for the traditional capacitor-current–proportional feedback damping technique stays positive in the frequency ranges (0, *f*_s_/6) and negative in the ranges (*f*_s_/6, *f*_s_/2), where *f*_s_/6 represents the damping region critical frequency *f*_crit_. Previous studies [24,27,28] inserted a first-order high-pass filter into the capacitor–current–feedback loop to compensate for the phase lag caused by digital delay, thereby widening the positive region (PVR) of the virtual resistor to (0, *f*_s_/3). Another study [19] adopted a second-order high-pass filter to achieve phase compensation exceeding 90°, and by adjusting the filter parameters, the *f*_crit_ of PVR can be valued within the range of (*f*_s_/3, *f*_s_/2). A researcher [29] adopted the capacitor-current proportional–integral positive feedback active damping technique, achieving a PVR that almost spans the entire frequency band with a *f*_crit_ of 0.48*f*_s_. For the grid current- feedback damping method, a study [30] implemented grid current feedback through a negative first-order high-pass filter to form a damping loop, increasing the damping zone’s *f*_crit_ to (*f*_s_/6, *f*_s_/3). Building upon this finding, another study [31] incorporated an additional first-order high-pass filter into the damping loop, extending the damping region to (*f*_s_/4, *f*_s_/2).

The PCC voltage unit feedforward technique can improve the current waveform fed into the power grid and effectively eliminate the background harmonics of the grid voltage. In addition, it can be considered equivalent to capacitor-voltage feedforward, with a certain damping effect [32,33,34,35,36] and a PVR of (0, *f*_s_/3). In-depth research [37] proposed a hybrid active damping method that combines the PCC voltage unit feedforward strategy with capacitor-current-feedback active damping, which can expand the PVR and increase *f*_crit_ to *f*_s_/2.

The PCC voltage unit feedforward strategy is usually used to suppress harmonics in the output current; its potential resonance suppression function, however, is used in the hybrid method adopted in [37] to expand the active damping region of resonance suppression. It is obvious that this method can enhance the resonance suppression capability without adding additional algorithms.

In order to further simplify the method in [37], this paper proposes a joint active damping method that combines the PCC voltage unit feedforward strategy with GCF active damping, using only the state variable feedback of the grid current. This method not only reduces the sampling of the capacitor current and saves three current transformers, but also simplifies control. In addition, this joint damping method can generate the dynamic damping range (DDR) by adjusting damping parameters that span the entire spectrum, even exceeding the Nyquist frequency. The experimental results show that this method has high robustness and can adapt to changes in grid impedance.

The rest of the content is arranged as follows: Section 2 examines the GCF’s and the PCC voltage unit feedforward’s active damping capabilities. In Section 3, a design approach for damping parameters is suggested, and a joint active damping system integrating GCF and PCC voltage unit feedforward is investigated. The efficiency of the proposed joint active damping technique is demonstrated in Section 4 through simulation and experimental results. This inquiry is concluded with a summary in Section 5.

## 2. Performance Analysis of PCC Voltage Unit Feedforward and GCF Damping Methods

### 2.1. System Description

**Figure 1:**
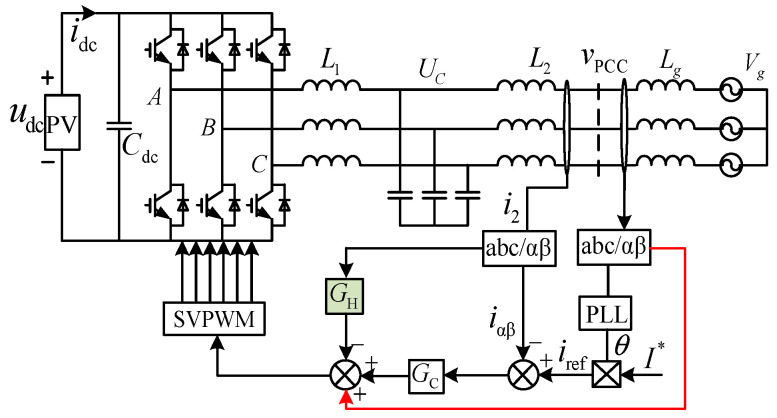
Block diagram of the control system for a three-phase *LCL* grid-connected inverter using the joint damping strategy.

The control system diagram of a three-phase *LCL*-type grid-connected inverter with a joint damping strategy is shown in Figure 1. The *LCL* filter is made up of filter capacitor *C*, grid side inductance *L*_2_, and inverter side inductance *L*_1_. The power grid is represented by an ideal voltage source and an inductance *L*_g_ linked in series at the PCC, with the inductance *L*_g_ being variable. To simplify the hardware structure and remove capacitor current sampling, the control system adopts a single current loop control strategy, i.e., the grid current feedback control loop. This current loop primarily tracks the given current *i*_ref_ by generating the grid-connected current *i*_g_, where *G*_C_ is a current controller with no static error tracking. To mitigate system resonance, the damping controller *G*_H_, a negative high-pass filter, is incorporated into another grid current feedback loop, operating as a damping loop (NFGCF). In addition, PCC voltage unit feedforward is used to reduce interference caused by background harmonics in the power grid voltage. The modulation reference signal for the space vector pulse width modulation (SVPWM) module is obtained by adding the outputs of the damping controller *G*_H_, the current controller *G*_C_, and *v*_PCC_ unit feedforward.

(1) $$G_{H}=\frac{-K_{C}s}{s+\omega_{d}},$$

where *K*_C_ denotes the high-pass filter gain and *ω*_d_ represents the cutoff angle frequency of the high-pass filter.

Taking into account the one-sample cycle delay required for modulation signal loading and the half-cycle delay introduced by the zero-order holder (ZOH) during signal sampling, a total delay link can be written as [15]

(2) $$G_{d}=e^{-1.5sT_{s}},$$

where *T*_S_ signifies the sampling period.

### 2.2. Active Damping Method

**Figure 2:**
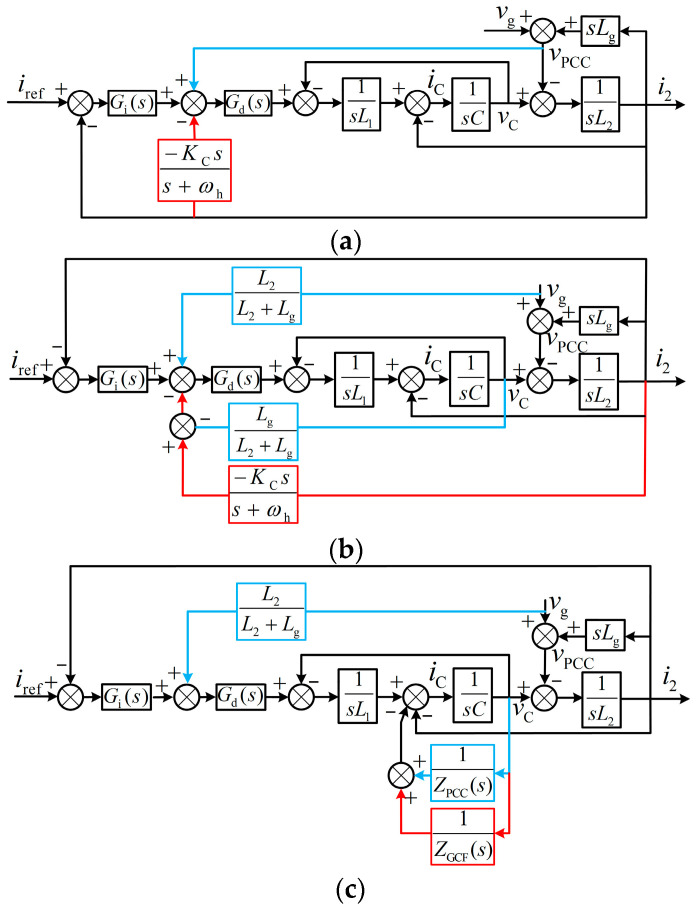
Control diagram of a grid-connected inverter system using NFGCF active damping and PCC voltage unit feedforward strategy. (**a**) Original block diagram and (**b**,**c**) equivalent block diagrams.

Figure 2a illustrates the corresponding control block diagram of the system shown in Figure 1 over a continuous range. Furthermore, the block diagrams depicted in Figure 2b,c are obtained through some simple equivalent transformations.

According to Figure 1, applying the superposition theorem, the representation of *v*_PCC_ is derived as follows:

(3) $$v_{\mathrm{PCC}}=\frac{L_{g}}{L_{g}+L_{2}}v_{C}+\frac{L_{2}}{L_{g}+L_{2}}v_{g},$$

where *v*_PCC_ comprises two components obtained by multiplying the capacitor voltage *v*_c_ and the grid voltage *v*_g_ using the weighting coefficient. According to Equation (3), by changing the feedback state variable *v*_PCC_ to *v*_c_ and *v*_g_, Figure 2a can be equivalent to Figure 2b. The grid voltage feedforward is utilized to mitigate the background harmonics introduced by grid voltage and has no impact on system stability [15]. Due to the contribution of capacitor voltage feedforward to filter resonance damping [16], *v*_PCC_ feedforward influences system damping and may also have a non-negligible impact on system stability.

**Figure 3:**
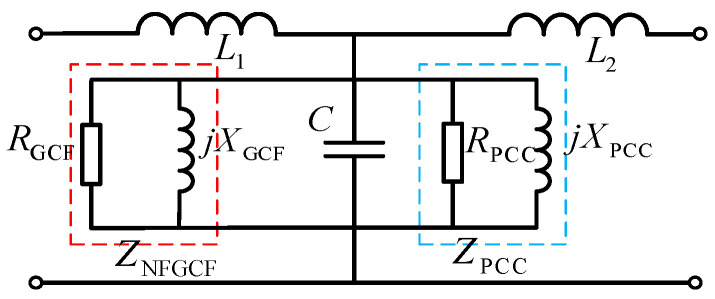
Equivalent circuit of joint damping.

In Figure 2b, first, convert the feedback state variable *i*_2_ of GCF into *v*_c_, then move the feedback nodes related to *v*_c_ from the input of *G*_d_(*s*) to the input of 1/(*sC*), and finally adjust the gains of the two feedback loops to obtain Figure 2c, and it is easy to derive

(4) $$Z_{\mathrm{PCC}}=-\frac{sL_{1}(L_{2}+L_{g})}{L_{g}}\frac{1}{G_{d}\left(s\right)},$$

(5) $$Z_{\mathrm{NFGCF}}=-\frac{s(s+\omega_{d})L_{1}(L_{2}+L_{g})}{K_{C}}\frac{1}{G_{d}\left(s\right)},$$

where the joint active damping method is equivalent to paralleling two virtual impedances, *Z*_PCC_ and *Z*_NFGCF_, at both ends of the capacitor (Figure 3).

Substituting *s* = *jω* into Equations (4) and (5) results in

(6) $$Z_{\mathrm{PCC}}(j\omega )=-\frac{j\omega (L_{2}+L_{g})L_{1}}{L_{g}}e^{1.5j\omega T_{s}}\underline{\underline{\Delta }}\;R_{\mathrm{PCC}}(\omega )\;//\;jX_{\mathrm{PCC}}(\omega ),$$

(7) $$R_{\mathrm{PCC}}(\omega )=-\frac{\omega L_{1}(L_{2}+L_{g})}{L_{g}}\frac{1}{\sin1.5\omega T_{s}},$$

(8) $$X_{\mathrm{PCC}}(\omega )=-\frac{\omega L_{1}(L_{2}+L_{g})}{L_{g}}\frac{1}{\cos1.5\omega T_{s}},$$

(9) $$\begin{array}{l} Z_{\mathrm{NFGCF}}(j\omega )=-\frac{\omega L_{1}(L_{2}+L_{g})(\omega -j\omega_{d})}{K_{C}}e^{1.5j\omega T_{s}}\\ \;\;\;\;\underline{\underline{\Delta }}\;R_{\mathrm{NFGCF}}(\omega )//jX_{\mathrm{NFGCF}}(\omega ), \end{array}$$

(10) $$R_{\mathrm{NFGCF}}(\omega )=\frac{\omega L_{1}(L_{2}+L_{g})(\omega^{2}+\omega_{d}^{2})}{K_{C}g_{R}(\omega )},$$

(11) $$X_{\mathrm{NFGCF}}(\omega )=\frac{\omega L_{1}(L_{2}+L_{g})(\omega^{2}+\omega_{d}^{2})}{K_{C}g_{X}(\omega )},$$

where

$$\left\{\begin{array}{l} g_{R}(\omega )=\sqrt{\omega^{2}+\omega_{d}^{2}}\sin(1.5\omega T_{s}+\theta )\\ g_{X}(\omega )=-\sqrt{\omega^{2}+\omega_{d}^{2}}\cos(1.5\omega T_{s}+\theta )\\ \theta =\arcsin(\omega /\sqrt{\omega^{2}+\omega_{d}^{2}}),\theta \in (0,\;\pi /2). \end{array}\right.$$

The two virtual impedances *Z*_PCC_ and *Z*_NFGCF_ are equivalent to parallel connections of a virtual resistor and a virtual reactance, respectively. The joint virtual resistance and reactance can be defined as

(12) $$\left\{\begin{array}{l} R_{\mathrm{eq}}=R_{\mathrm{NFGCF}}//R_{\mathrm{PCC}}\\ X_{\mathrm{eq}}=X_{\mathrm{NFGCF}}//X_{\mathrm{PCC}}. \end{array}\right.$$

Following Figure 2c, the system’s open-loop gain expression can be derived as follows:

(13) $$T\left(s\right)=\frac{G_{d}\left(s\right)G_{i}\left(s\right)}{sL_{1}(L_{2}+L_{g})C}\cdot \frac{1}{s^{2}+\frac{1}{Z_{\mathrm{eq}}\left(s\right)C}\cdot s+\omega_{r}^{2}},$$

where *ω*_r_ denotes the resonance frequency of the *LCL* filter as follows:

(14) $$\omega_{r}=2\pi f_{r}=\sqrt{\frac{L_{1}+L_{2}+L_{g}}{L_{1}(L_{2}+L_{g})C}}.$$

The denominator of the quadratic term in (13) is assumed to be

(15) $$d\left(s\right)=s^{2}+\frac{1}{Z_{\mathrm{eq}}\left(s\right)C}\cdot s+\omega_{r}^{2}.$$

Substituting *s* = *jω*_r_′ into Equation (15) yields

(16) $$d(j{\omega_{r}}^{\prime })=-{\omega_{r}}^{\prime 2}+\frac{{\omega_{r}}^{\prime }}{X_{\mathrm{eq}}({\omega_{r}}^{\prime })C}+\omega_{r}^{2}+j\frac{{\omega_{r}}^{\prime }}{R_{\mathrm{eq}}({\omega_{r}}^{\prime })C},$$

where *ω*_r_′ signifies the system resonance frequency. Letting the real part of *d*(*jω*_r_′) equal zero [38] derives

(17) $$-{\omega_{r}}^{\prime 2}+\frac{{\omega_{r}}^{\prime }}{X_{\mathrm{eq}}({\omega_{r}}^{\prime })C}+\omega_{r}^{2}=0.$$

By solving Equation (17), the system resonance frequency can be obtained as follows:

(18) $${f_{r}}^{\prime }=\frac{{\omega_{r}}^{\prime }}{2\pi }=\left\{\begin{array}{l} \frac{1}{2\pi }\sqrt{\frac{L_{1}+L_{2}+L_{g}}{\left(C+C_{\mathrm{eq}})L_{1}(L_{2}+L_{g}\right)}}\;\;\;X_{\mathrm{eq}}=C_{\mathrm{eq}}\\ \frac{1}{2\pi }\sqrt{\frac{L_{1}+L_{2}+L_{g}}{CL_{1}(L_{2}+L_{g})}+\frac{1}{CL_{\mathrm{eq}}}}\;\;\;X_{\mathrm{eq}}=L_{\mathrm{eq}} \end{array}\right.\;.$$

**Figure 4:**
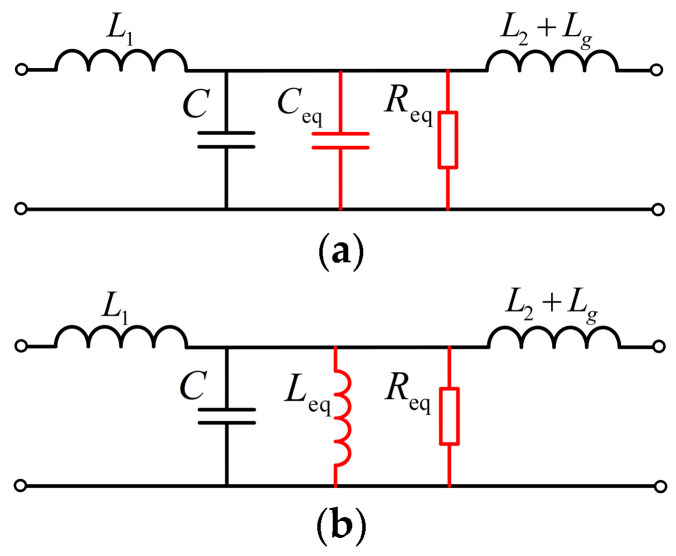
Active damping equivalent circuit. *X*_eq_ behaves as the (**a**) capacitive reactance and (**b**) inductive reactance.

Equation (18) reveals that the equivalent reactance *X*_eq_ can be depicted as capacitive and inductive reactances (Figure 4). Comparing Equation (18) with Equation (14) (the filter resonance frequency formula), when *X*_eq_ is the capacitive reactance, the system resonance frequency *f*_r_′ < filter resonance frequency *f*_r_. When *X*_eq_ is the inductive reactance, *f*_r_′ > *f*_r_. In addition, the parallel equivalent *R*_eq_ at both ends of the capacitor dampens the system’s resonance peak, and the sign of *R*_eq_(*f*_r_′) determines the number of the open-loop RHP poles [17,18].

### 2.3. NFGCF Damping and PCC Voltage Feedforward Damping

**Figure 5:**
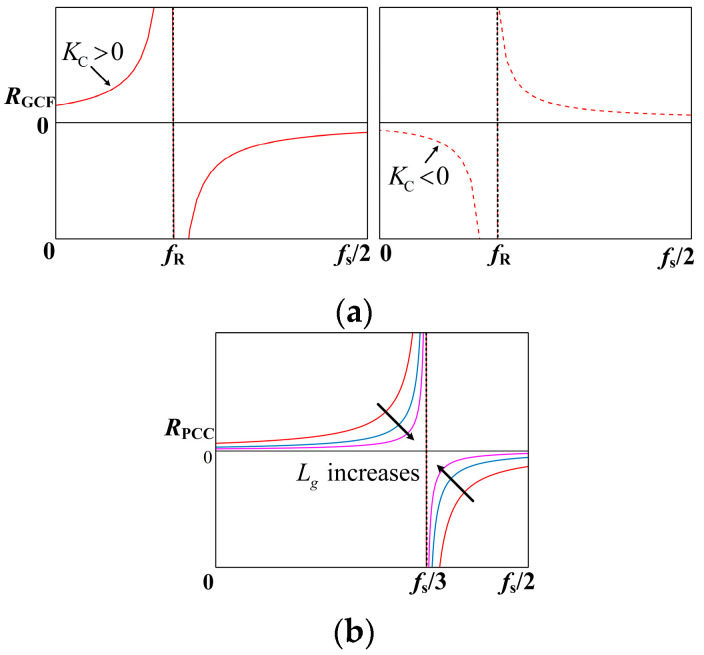
Frequency characteristics of (**a**) *R*_NFGCF_ with different *K*_c_ and (**b**) *R*_PCC_.

The frequency characteristics of *R*_NFGCF_ and *R*_PCC_ are plotted in Figure 5a and 5b, respectively, based on Equations (7) and (10). In Figure 5a, when *K*_C_ > 0, *R*_NFGCF_ is positive in the frequency range (0, *f*_R_) and negative in the frequency range (*f*_R_, *f*_s_/2), where *f*_R_ represents the critical frequency in the range (*f*_s_/6, *f*_s_/3) [31]. When *K*_C_ < 0, the frequency characteristics of *R*_NFGCF_ exhibit an opposite trend. A negative *R*_NFGCF_ at *f*_r_’ triggers the system to generate a pair of open-loop RHP poles, i.e., *p* = 2, yielding a non-minimum phase behavior of the system.

*R*_PCC_ is associated with *L*_g_, as derived from Equation (7). When *L*_g_ = 0, *R*_PCC_ approaches infinity, rendering the *R*_PCC_ branch in Figure 4 equivalent to an open circuit with no damping effect. As *L*_g_ increases, *R*_PCC_ gradually diminishes, and the damping effect gradually increases. Based on the frequency characteristics of *R*_PCC_ (Figure 5b), the positive region of *R*_PCC_ spans (0, *f*_s_/3) and the negative region covers (*f*_s_/3, *f*_s_/2], irrespective of the value of *L*_g_. When *f*_r_′ falls within the negative range of *R*_PCC_, two open-loop RHP poles appear.

**Figure 6:**
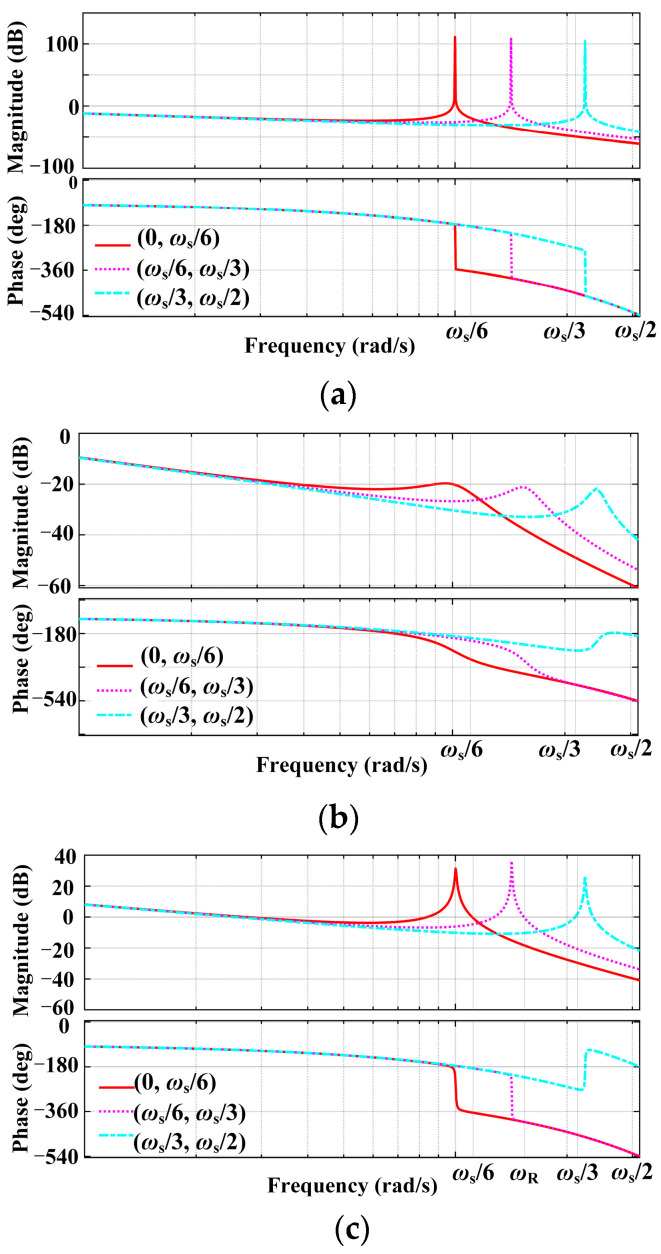
Open-loop gain Bode plots of the system when *G*_i_(*s*) = 1 with (**a**) no active damping GCF, (**b**) PCC voltage unit feedforward, and (**c**) NFGCF active damping.

Figure 6 illustrates the system’s open-loop gain Bode plots with no active damping GCF, PCC voltage unit feedforward, and NFGCF active damping scenarios. In each Bode plot, three possible scenarios are considered based on the value region of *ω*_r_′.

In Figure 6a, the system adopts the GCF loop control strategy (without an active damping loop). Due to the inherent characteristics of the current loop, the system exhibits damping within the frequency range (*ω*_s_/6, *ω*_s_/2). When *ω*_r_′ falls within (0, *ω*_s_/6), the phase plots pass through −180° once at *ω*_r_′ = *ω*_r_, resulting in *ω*_r_ exhibiting an infinite resonance peak, rendering the system unstable, regardless of controller parameter adjustment. When *ω*_r_′ lies within (*ω*_s_/6, *ω*_s_/2), the phase plots cross over −180° once at *ω*_r_′ = *ω*_r_, and the corresponding amplitude at *ω*_r_ avoids the resonance peak. Achieving system stability in this case requires selecting the appropriate controller parameters. According to the Nyquist stability criterion, the second instance can achieve system stability by modifying the proportional coefficient of the current controller, whereas the first situation requires additional active damping.

The open-loop gain Bode plot of the PCC voltage unit feedforward strategy is shown in Figure 6b. It is noticed that the phase plots decline monotonically over the whole frequency range, whereas the resonance peak of the amplitude is greatly reduced when *ω*_r_′ is in (0, *ω*_s_/3). On the other hand, a secondary crossing at −180° takes place when *ω*_r_′ lies inside (*ω*_s_/3, *ω*_s_/2), resulting in non-minimum phase behavior and system instability. To ensure system stability, more stringent requirements for the gain margin must be satisfied by modifying the current controller’s specifications.

The NFGCF active damping scenario (*K*_C_ > 0) is illustrated in Figure 6c. When *ω*_r_′ is in (0, *ω*_R_), the amplitude resonance peak is moderately suppressed, and further resonance suppression can be achieved by tuning the gain *K*_C_ of the high-pass filter. When *ω*_r_′ falls within (*ω*_R_, *ω*_s_/2), *R*_eq_ (*f*_r_′) becomes negative, and a pair of open-loop RHP poles appear. The phase plots exhibit two −180° crossovers. According to the Nyquist stability criterion, a stricter gain margin requirement must be met to ensure system stability. In addition, *ω*_r_′ is influenced by *L*_g_; therefore, large-scale variations in *L*_g_ can induce system instability.

Both NFGCF and PCC voltage unit feedforward can produce damping effects, but they have fixed negative damping frequency ranges. Therefore, they are more vulnerable to fluctuations in grid impedance in weak grid scenarios, causing system instability. To address this issue, the system’s open-loop frequency characteristics must meet more strict gain margin criteria, which requires changes to the damping loop’s control parameters.

## 3. Joint Active Damping

### 3.1. Damping Characteristics of Joint Active Damping

**Figure 7:**
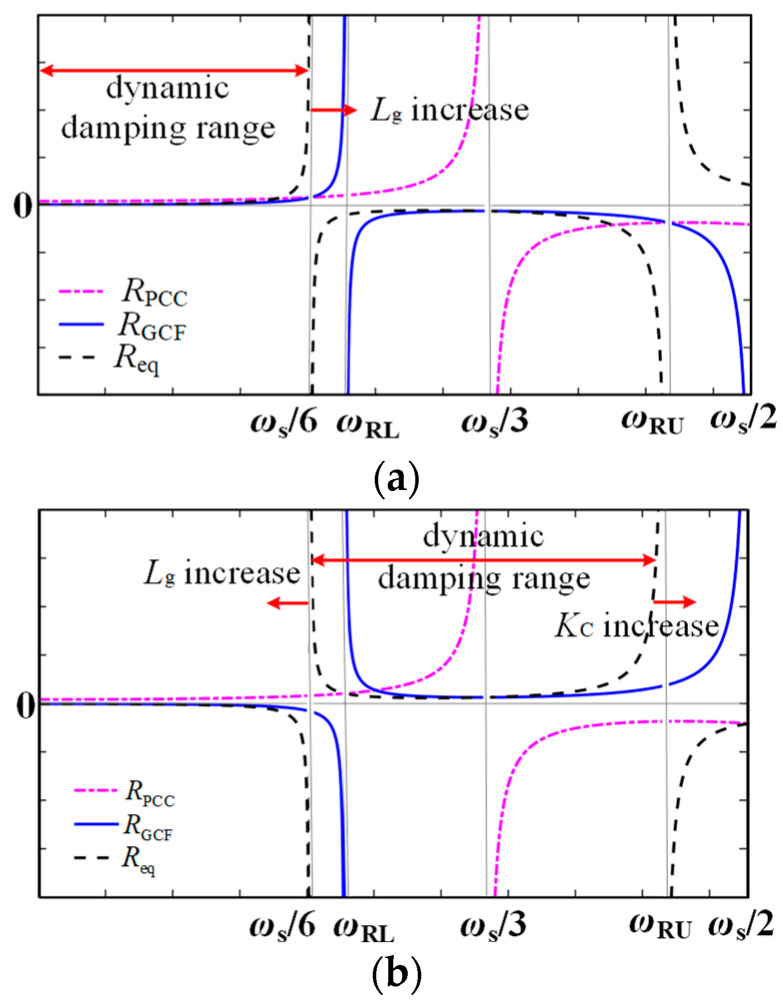
Frequency characteristics of *R*_eq_ at (**a**) *K*_C_ > 0 and (**b**) *K*_C_ < 0.

The background harmonics of the grid voltage introduce harmonics into the grid-connected current, leading to current waveform distortion. Introducing PCC voltage unit feedforward can suppress these harmonics in the grid-connected current. Both NFGCF and PCC voltage unit feedforward approaches can suppress resonance, making it worthwhile to explore their joint effect. Figure 7 illustrates the frequency characteristics of *R*_eq_.

In Figure 7a, when *L*_g_ = 0 following Equation (3), the PCC voltage equates to the grid voltage. With no capacitor voltage component, the voltage feedforward exhibits no damping effect, and only *R*_NFGCF_ provides a damping effect. *ω*_RL_ and *ω*_RU_ represent the upper and lower boundary frequencies at which *R*_eq_(*f*_r_′) becomes negative. For *K*_C_ > 0, when *ω*_r_′ *> ω*_RL_, *R*_NFGCF_ < 0; and when *ω*_r_′ < *ω*_RL_, *R*_NFGCF_ > 0. When *L*_g_ is not zero, *R*_PCC_ contributes to the overall damping effect. In this case, *R*_eq_ is negative within (*ω*_s_/3, *ω*_s_/2) because *R*_NFGCF_ and *R*_PCC_ are negative in the same frequency region. As *L*_g_ increases from zero to *L*_gmax_, the lower boundary of the PVR of *R*_eq_ remains constant at zero, while the upper boundary values increase with increasing *L*_g_, without exceeding *ω*_s_/3.

In Figure 7b, when *L*_g_ = 0, only *R*_NFGCF_ contributes to the damping effect. For *K*_C_ < 0, when *ω*_RU_ > *ω*_r_′ > *ω*_RL_, *R*_NFGCF_ > 0, and when *ω*_r_′ < *ω*_RL_, *R*_NFGCF_ < 0. When *L*_g_ is not zero, *R*_PCC_ participates in the overall damping effect. As *L*_g_ increases from zero to *L*_gmax_, the upper and lower boundaries of the PVR of *R*_eq_ shift leftward simultaneously. However, with the gradual increase in *K*_C_, the upper and lower boundaries of the PVR of *R*_eq_ shift to the right concurrently, forming a DDR with a width close to *ω*_s_/3. In addition, the upper boundary of this DDR can surpass the Nyquist frequency *ω*_s_/2, and the lower boundary value can reach 0 Hz.

In practical applications, the filter’s resonance frequency is typically *ω*_r_ > *ω*_s_/6. Therefore, the joint damping method combining PCC voltage unit feedforward and NFGCF (*K*_C_ < 0) active damping can establish a DDR. The upper and lower boundaries of the DDR can adapt to variations in *L*_g_ and can be controlled by adjusting *K*_C_ in NFGCF. To address significant changes in *L*_g_, a suitable *K*_C_ is chosen to achieve robust system control.

### 3.2. Arch on PVR

Determining the upper and lower boundaries of the PVR involves finding their functional relationship with the control parameter *K*_C_ in NFGCF and *L*_g_. Following Equation (12), the equivalent resistance *R*_eq_ of the joint damping approach can be derived as follows:

(19) $$R_{\mathrm{eq}}=\frac{-L_{1}(L_{2}+L_{g})\omega \sqrt{\omega^{2}+\omega_{d}^{2}}}{K_{C}\sin(1.5T_{S}\omega +\theta )-L_{g}\sqrt{\omega^{2}+\omega_{d}^{2}}\sin(1.5T_{S}\omega )}.$$

From Figure 7, when *R*_eq_ at the boundary frequency approaches infinity, the denominator of Equation (19) can be set to zero for boundary frequency determination, yielding

(20) $$K_{C}\sin(1.5T_{s}\omega +\theta )-L_{g}\sqrt{\omega^{2}+\omega_{d}^{2}}\sin(1.5T_{s}\omega )=0$$

Equation (20) reveals that after establishing *K*_C_ and *ω*_d_, the boundary frequency can be determined using *L*_g_. Ensuring *R*_eq_(*f*_r_′) > 0 necessitates maintaining *ω*_r_′(*L*_g_) in Equation (18) within the upper and lower boundary frequencies derived from Equation (20). After a simple mathematical transformation, we reformulate Equation (17) regarding *ω*_r_′ as

(21) $$\begin{array}{l} K_{C}\cos(1.5T_{s}{\omega_{r}}^{\prime }+\theta )-L_{g}\cos(1.5T_{s}{\omega_{r}}^{\prime })\sqrt{{\omega_{r}}^{\prime 2}+\omega_{d}^{2}}\;\;\;\;\;.\\ +(L_{1}+L_{2}+L_{g})\sqrt{{\omega_{r}}^{\prime 2}+\omega_{d}^{2}}-{\omega_{r}}^{\prime 2}CL_{1}(L_{2}+L_{g})\sqrt{{\omega_{r}}^{\prime 2}+\omega_{d}^{2}}=0 \end{array}$$

Obtaining an analytical solution for the boundary frequency is challenging; therefore, it can be substituted by utilizing the data in Table 1 and combining Equations (20) and (21) to construct a graph. In this context, *L*_g_ fluctuates from 0 to 0.1 p.u., corresponding to *L*_gmax_ = 2 mH.

**Figure 8:**
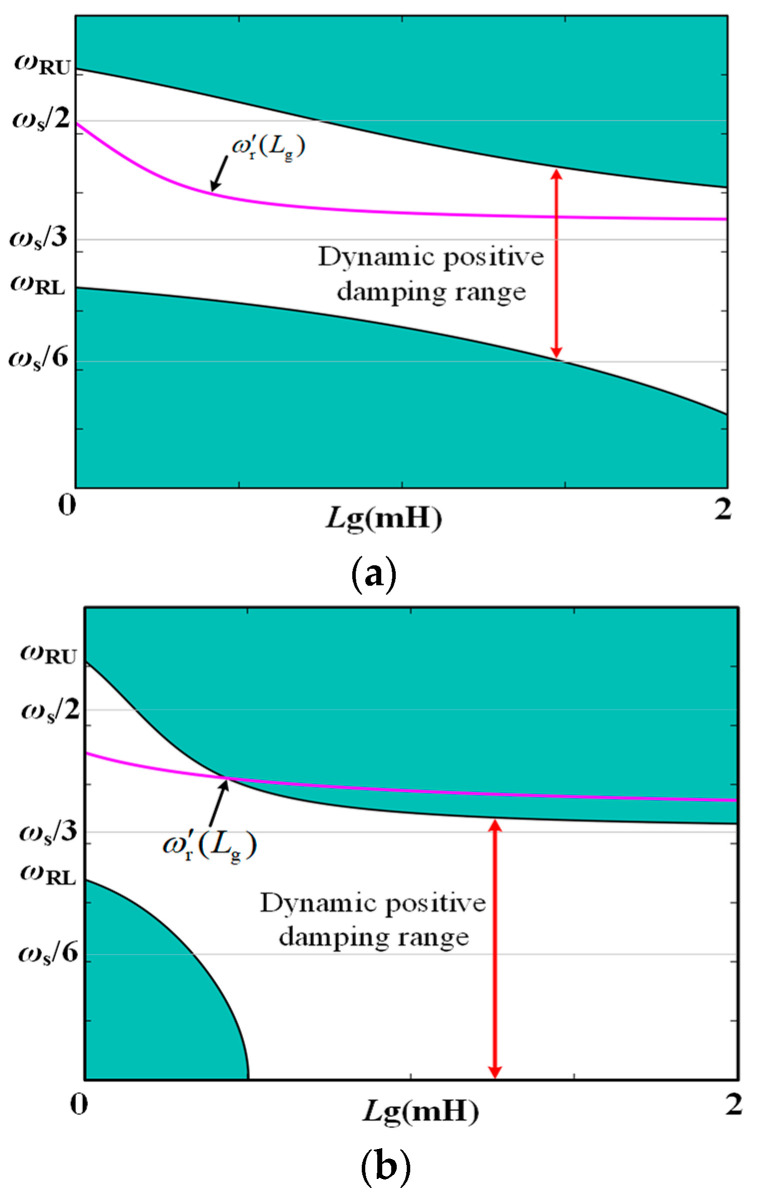
Relationship between the DDR and *L*_g_ at (**a**) *K*_C_ = −40, *ω*_d_ = 6000π and (**b**) *K*_C_ = −10, *ω*_d_ = 6000π.

Figure 8 illustrates the relationship between the DDR and *L*_g_ under various *K*_C_ values. It also presents the curve depicting the system’s resonance frequency *ω*_r_′(*L*_g_) at different *L*_g_ values. The following conclusions can be drawn from the figure:

1. When *L*_g_ = 0, only *R*_NFGCF_ contributes to damping, with a PVR of (*ω*_RL_, *ω*_RU_). *ω*_RL_ exceeds *ω*_s_/6 but falls below *ω*_s_/3, while *ω*_RU_ exceeds the Nyquist frequency *ω*_s_/2.
2. When *L*_g_ > 0, *R*_NFGCF_ and *R*_PCC_ provide damping. With rising *K*_C_ and *L*_g_, the upper and lower boundaries of the DDR shift downward. When *K*_C_ approaches zero, *R*_NFGCF_ has minimal impact, and *R*_PCC_ operates independently, leading to a DDR of (0, *ω*_s_/3) (Figure 8b).
3. From Figure 8a,b, when *K*_C_ remains constant, ω_r_′ decreases monotonically with varying *L*_g_. When *K*_C_ = −40, ω_r_′ consistently falls within the DDR. However, when *K*_C_ = −10, as *L*_g_ increases, a portion of the *ω*_r_′(*L*_g_) curve exceeds the DDR, indicating an inappropriate *K*_C_ selection.

### 3.3. Parameter Design

**Figure 9:**
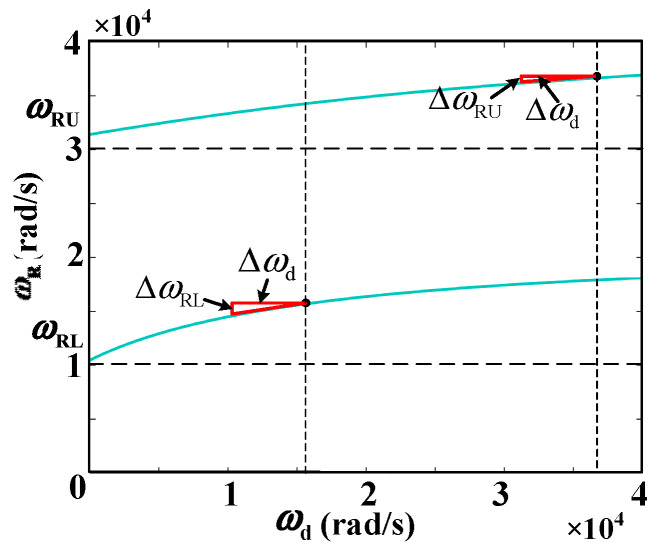
The relationship between *ω*_RL_ and *ω*_RU_ with *ω*_d_.

The system control parameters encompass the current loop and damping loop control parameters, *K*_C_ and *ω*_d_, respectively. The preceding analysis reveals that when *L*_g_ = 0, *R*_NFGCF_ yields a damping effect, and the initial boundary frequencies *ω*_RL_ and *ω*_RU_ are solely contingent on *ω*_d_. According to Equation (20), sin(1.5*ωT*_s_ + *θ*) = 0 holds true and simplifies to yield

(22) $$1.5\omega T_{s}+\arcsin(\frac{\omega }{\sqrt{\omega^{2}+\omega_{d}^{2}}})=\pi \;\mathrm{or}\;2\pi ,$$

where *ω* = *ω*_RL_ or *ω*_RU_. Following Equation (22), we plot curves depicting the relationship between the initial boundary frequencies of the DDR (*ω*_RL_ and *ω*_RU_) and *ω*_d_ (Figure 9). The initial boundary frequencies increase monotonically with increasing *ω*_d_. However, the slopes of these curves are relatively small. In proximity to a fixed frequency point, the alterations in initial boundary frequencies caused by a larger Δ*ω*_d_ (Δ*ω*_RL_ and Δ*ω*_RU_) are insignificant. Consequently, Δ*ω*_d_ can be determined first, and the DDR can be adjusted by fine-tuning the parameter *K*_C_. The optimal performance in control systems can be achieved by ensuring that *ω*_d_ is near *ω*_r_ [30]. Therefore, in this study, we select *ω*_d_ = *ω*_r_.

Based on the above analysis and the choice of *ω*_d_, the DDR relies on the *K*_C_. Selecting an appropriate *K*_C_ can ensure that the system’s resonance frequency remains within the DDR, achieving minimum phase behavior. However, these settings simplify meeting the stability requirements for the system. For the given phase margin and amplitude margin, the design process of controllers *G*_i_(*s*) and *K*_C_ is outlined as follows.

Employing a quasi-proportional resonant PR controller can achieve reduced steady-state errors at the fundamental frequency. Therefore, in our study, we utilize a PR controller, expressed as follows:

(23) $$G_{\mathrm{PR}}(s)=K_{P}+\frac{2K_{r}\omega_{i}s}{s^{2}+2\omega_{i}s+\omega_{0}^{2}},$$

where *K*_P_ and *K*_r_ denote the proportional and resonance coefficients, respectively; *ω*_o_ signifies the fundamental frequency; and *ω*_i_ represents the −3 dB bandwidth near the resonance frequency, i.e., the gain at *ω*_o_ ± *ω*_i_ is 0.707 *K*_r_. To ensure the adequate gain of the PR regulator near the fundamental frequency during grid frequency fluctuations, we set the parameters to *ω*_i_ = 2πΔ*f* = πrad/s [12]. Compared with PI controllers, PR controllers can boost the fundamental gain and mitigate steady-state tracking errors at fundamental frequency *ω*_o_. However, a significant negative phase shift is introduced in the frequency region higher than the fundamental frequency, particularly near the fundamental frequency, impacting the system’s phase margin. To address this issue, the system’s cutoff frequency *ω*_c_ needs to be substantially higher than *ω*_o_. Therefore, based on (23), the gains of the PR controller at *ω*_o_ and *ω*_c_ are derived to be *G*_i_(*jω*_o_) = *K*_P_ + *K*_r_ and *G*_i_(*jω*_c_) = *K*_P_ + 2*K*_r_*ω*_i_/*jω*_c_. Typically, when analyzing frequency characteristics in the frequency range higher than *ω*_c_, the PR controller can be approximated as a proportional coefficient *K*_P_, expressed as *K*_P_ ≈ *ω*_c_(*L*_1_ + *L*_2_). In practice, *ω*_c_ is often selected to be 4% of *ω*_s_. Considering that *L*_g_ is nonzero and *K*_P_ employs the aforementioned formula, the actual *ω*_c_ may not satisfy the design requirements. When selecting the system sampling frequency of 10 kHz, *ω*_c_ in the expression of *K*_P_ slightly exceeds the design requirements. Therefore, *ω*_c_ is set to 6% of *ω*_s_, equivalent to 600 Hz. In addition, the selection range of phase margin PM is 30~60° [39], and the choice of *K*_r_ should consider the negative phase shift introduced by the PR controller to meet the requirements of phase margin PM.

**Figure 10:**
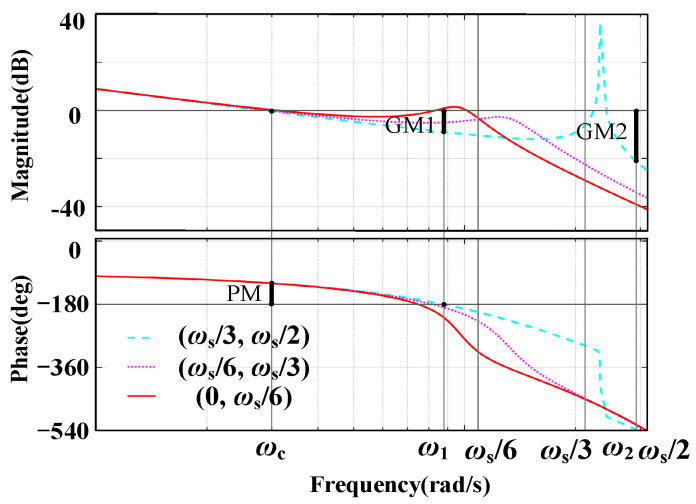
Open-loop gain Bode plot of joint damping approach.

If the system satisfies *R*_eq_ (*f*_r_′) > 0, the minimum phase system can be guaranteed, resulting in zero open-loop RHP poles. Employing our proposed joint damping approach, the open-loop gain Bode plot of the system with *ω*_r_′ across various frequency ranges is shown in Figure 10. According to the Nyquist stability criterion, *N*(+) − *N*(−) = 0 must be satisfied to ensure system stability, where *N*(+) and *N*(−) denote the numbers of the phase-frequency curve’s positive and negative crossings over −180° and −540° lines, respectively. The figure demonstrates that the phase-frequency curve of the open-loop system monotonically decreases, crossing −180° and −540° at *ω*_1_ and *ω*_2_, respectively, indicating *N*(+) = 0. In addition, only when GM_1_ > 0 and GM_2_ > 0 are satisfied simultaneously can the negative crossing of the phase frequency curve at the −180° and −540° lines be negated, resulting in N(−) = 0.

To meet the two requirements of the amplitude margin, a constraint relationship between GM_1_ and GM_2_ regarding *K*_C_ needs to be determined. By substituting *s* = *jω*_1_ and *s* = *jω*_2_ into Equation (13), and assuming GM_1_ = −20lg|T(*jω*_1_)| and GM_2_ = −20lg |T(*jω*_2_)|, the boundary value of *K*_C_ constrained by GM_1_ and GM_2_ can be obtained.

The crossing frequencies *ω*_1_ and *ω*_2_ satisfy the following Equations:

(24) $${\mathrm{GM}}_{1}+20\mathrm{lg}(\frac{K_{P}({\omega_{1}}^{2}+{\omega_{d}}^{2})}{\omega_{1}\sqrt{a^{2}+b^{2}}})=0,$$

(25) $${\mathrm{GM}}_{2}+20\mathrm{lg}(\frac{K_{P}({\omega_{2}}^{2}+{\omega_{d}}^{2})}{\omega_{2}\sqrt{a^{2}+b^{2}}})=0,$$

(26) $$\pi /2-1.5\omega T_{s}-\arctan(b/a)=0,\;\omega =\omega_{1},$$

(27) $$3\pi /2-1.5\omega T_{s}-\arctan(b/a)=0,\;\omega =\omega_{2},$$

where

$$\begin{array}{l} a=c+(K_{C}-\omega_{d}L_{g})\omega_{d}\cos(1.5\omega T_{s})-\omega^{2}L_{g}\cos(1.5\omega T_{s})\;\\ -L_{g}\omega \omega_{d}\sin(1.5\omega T_{s})-(K_{C}-\omega_{d}L_{g})\omega \sin(1.5\omega T_{s}) \end{array},$$

$$\begin{array}{l} b=\omega^{2}L_{g}\sin(1.5\omega T_{s})-L_{g}\omega \omega_{d}\cos(1.5\omega T_{s})\;\\ -(K_{C}-\omega_{d}L_{g})\omega \cos(1.5\omega T_{s})-(K_{C}-\omega_{d}L_{g})\omega_{d}\sin(1.5\omega T_{s}) \end{array},$$

$$c=L_{1}(L_{2}+L_{g})C(\frac{L_{1}+L_{2}+L_{g}}{L_{1}(L_{2}+L_{g})C}-\omega^{2})(\omega^{2}+{\omega_{d}}^{2}).$$

To achieve the stable operation of the system, it is essential to meet the phase margin PM requirement. By substituting *s* = *jω*_c_ into Equation (13), the phase margin PM can be defined as

(28) $$\mathrm{PM}=\pi -\pi /2-1.5\omega T_{s}-\arctan(b/a)+∠G_{\mathrm{PR}}(s).$$

### 3.4. Design Program

**Figure 11:**
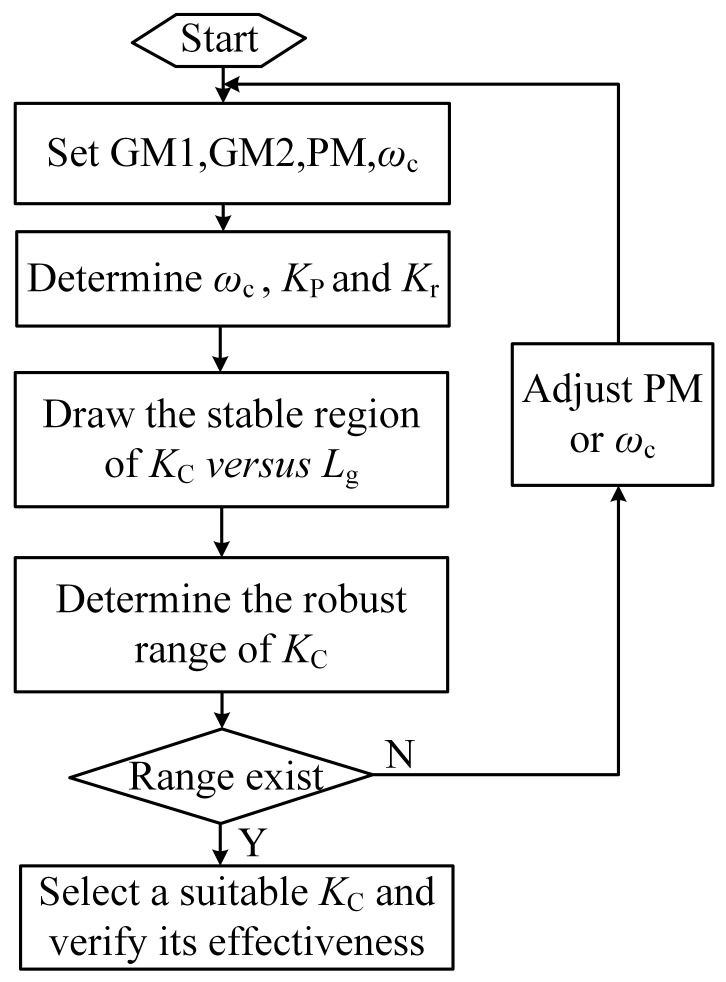
Program flowchart.

Following the above analysis, the design program is outlined as depicted in Figure 11, and the design steps are detailed as follows.

1. Given *ω*_c_ and PM, the appropriate controller parameters *K*_P_ and *K*_r_ are determined.
2. Based on the specified GM_1_, GM_2_, and selected *K*_P_, Equations (24)–(28) are used to determine the achievable range of *K*_c_ for varying *L*_g_.
3. The *K*_C_ value from the value range available is chosen to ensure robust control of the system. If stringent requirements for GM_1_, GM_2_, and PM exist and there is no robust range of *K*_C_, the design steps are executed from scratch by adjusting *ω*_c_ and PM.
4. A suitable *K*_C_ is selected to validate the efficacy of the proposed method.

This design program employs the equipment parameters listed in Table 1, where *ω*_c_ = 6% of *ω*_s_, PM = 30°, GM_1_ = GM_2_ = 3 dB, *K*_P_ = 3.4, and *K*_r_ = 307. Following Equations (21) and (23), the constraint relationship between *K*_C_, *ω*_r_′, and *L*_g_ is established, represented in three-dimensional graphics.

**Figure 12:**
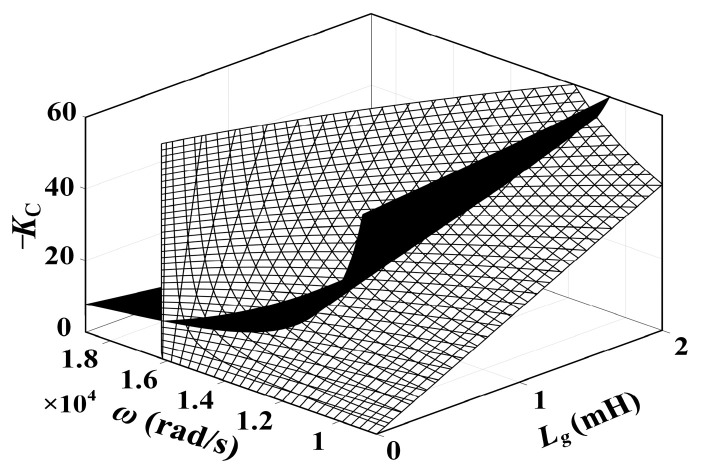
The constraint relationship between *K*_C_ versus *ω*_r_′ and *L*_g_.

The two curved surfaces in Figure 12 represent the constraint relationship between *K*_C_, *ω*_r_′, and *L*_g_, where the intersecting line indicates the common solution of Equations (21) and (24).

**Figure 13:**
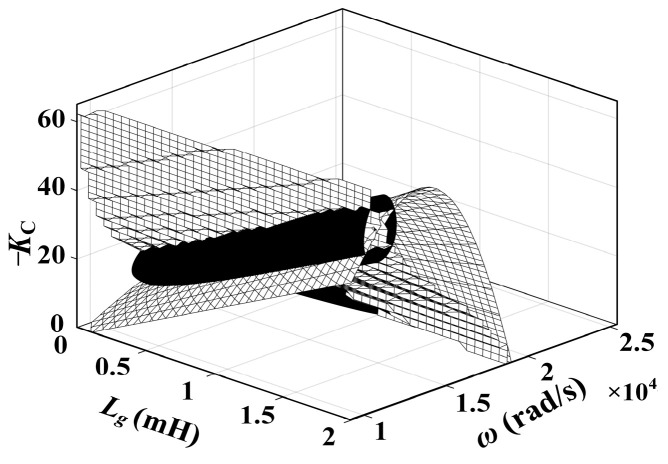
The constraint relationship between *K*_C_ and *L*_g_ under the GM_1_ and GM_2._ requirements.

Since obtaining an analytical solution for the crossing frequencies *ω*_1_ and *ω*_2_ is challenging, the three-dimensional surfaces are depicted separately, and the relationship between *K*_C_ and *L*_g_ is determined using the intersection projection approach. Figure 13 illustrates the constraint relationship between *K*_C_ and *L*_g_ while adhering to GM_1_ and GM_2_ requirements, based on Equations (24)–(27).

**Figure 14:**
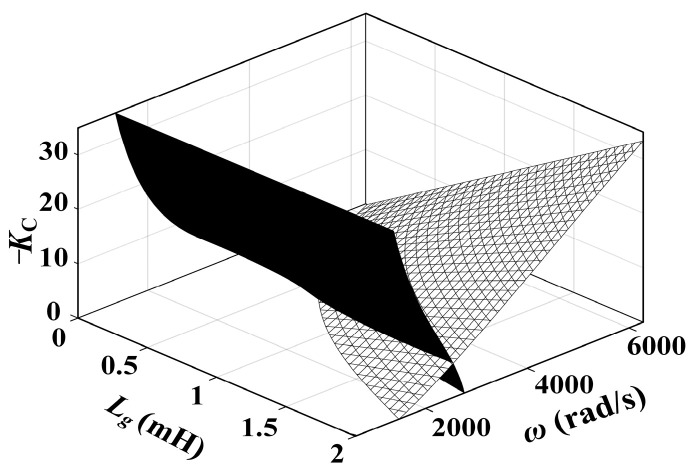
The constraint relationship between *K*_C_ and *L*_g_ under the PM requirements.

According to Equation (24) (GM_1_ = 0 dB) and Equation (28), the constraint relationship between *K*_C_ and *L*_g_ under the PM requirements can be plotted, as shown in Figure 14.

**Figure 15:**
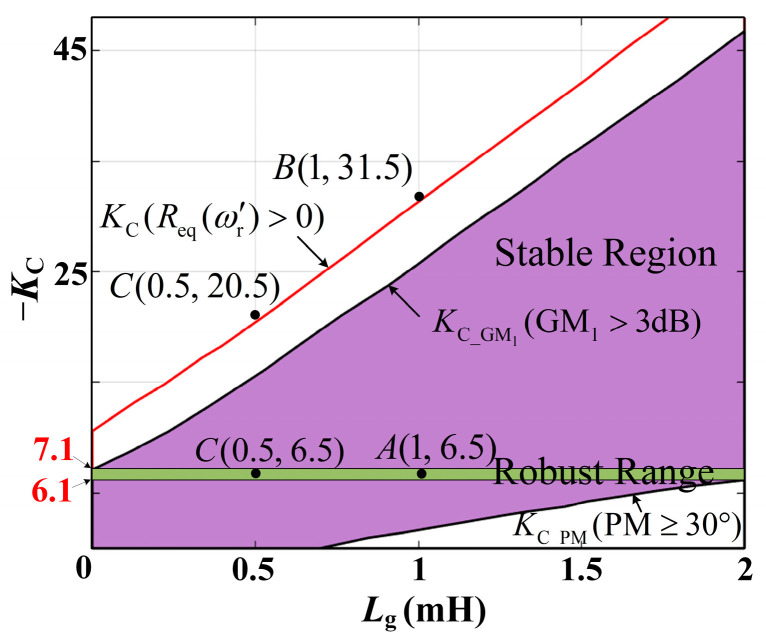
Value range of *K*_C_ against *L*_g_ variation.

By projecting the intersecting lines of the surfaces in Figure 12, Figure 13 and Figure 14 onto the *K*_C_–*L*_g_ plane and synthesizing the above constraint curve, the final constraint relationship boundary between *K*_C_ and *L*_g_ can be obtained (Figure 15). The graph reveals that the constraint boundary for GM_2_ is missing because the requirement of GM_2_ > 3 dB is consistently satisfied within the depicted value range of *K*_C_ and *L*_g_. In addition, when *L*_g_ varies from 0 to 2 mH (corresponding to a short-circuit ratio, SCR of 10), the robust value range of *K*_C_ is [−7.1, −6.1].

To validate the effectiveness of the design steps, Points A (*L*_g_ = 1 mH and *K*_C_ = −6.5), B (*L*_g_ = 1 mH and *K*_C_ = −31.5), C (*L*_g_ = 0.5 mH and *K*_C_ = −6.5), and D (*L*_g_ = 0.5 mH and *K*_C_ = −20.5) are shown in Figure 15 as examples for comparative experiments.

## 4. Simulation and Experimental Verification

### 4.1. Simulation Results

We simulate and compare the four sets of *K*_C_–*L*_g_ values represented by Points A, B, C, and D in Figure 15. During simulation, all circuit and control parameters remain consistent except for variations in *K*_C_ and *L*_g_.

**Figure 16:**
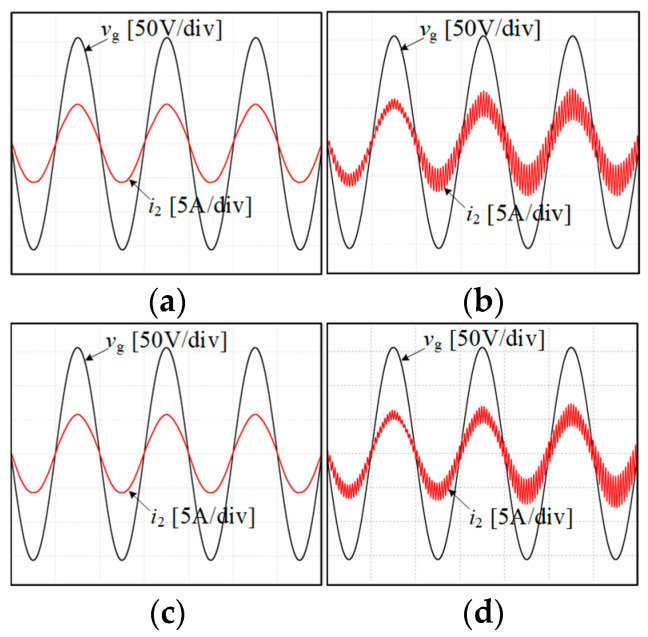
Simulation of a grid-connected inverter system under different grid impedance and *K*_C_. (**a**) Point A: *L*_g_ = 1 mH, *K*_C_ = −6.5. (**b**) Point B: *L*_g_ = 1 mH, *K*_C_ = −31.5. (**c**) Point C: *L*_g_ = 0.5 mH, *K*_C_ = −6.5. (**d**) Point D: *L*_g_ = 0.5 mH, *K*_C_ = −20.5.

**Figure 17:**
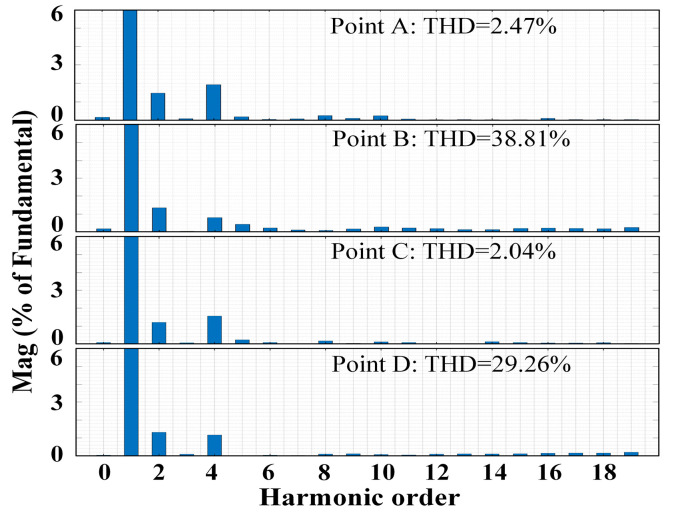
An analysis of the waveform total harmonic distortion (THD) of the grid current under four different scenarios.

Figure 16 presents simulation-based comparative results of different points: Point A (*L*_g_ = 1 mH, *K*_C_ = −6.5), Point B (*L*_g_ = 1 mH, *K*_C_ = −31.5), Point C (*L*_g_ = 0.5 mH, *K*_C_ = −6.5), and Point D (*L*_g_ = 0.5 mH, *K*_C_ = −20.5). Figure 16a,b indicate when the grid impedance *L*_g_ = 1 mH, the measured grid current waveform remains stable at *K*_C_ = −6.5. However, at *K*_C_ = −31.5, the waveform oscillates and the system becomes unstable. Figure 16c,d indicate that when the grid impedance *L*_g_ = 0.5 mH, the measured grid current waveform oscillates and the system becomes unstable. These simulation results align with the conclusions presented in Figure 15. Figure 17 presents a THD analysis of the grid current waveform under four different scenarios. Following the IEEE 519-2014 standard [40], the single harmonic and total harmonic distortion (THD) in the simulation waveform results in Figure 16a,c satisfy the requirements. By contrast, the simulation waveform results of Figure 16b,d exhibit severe distortion.

### 4.2. Experimental Results

**Figure 18:**
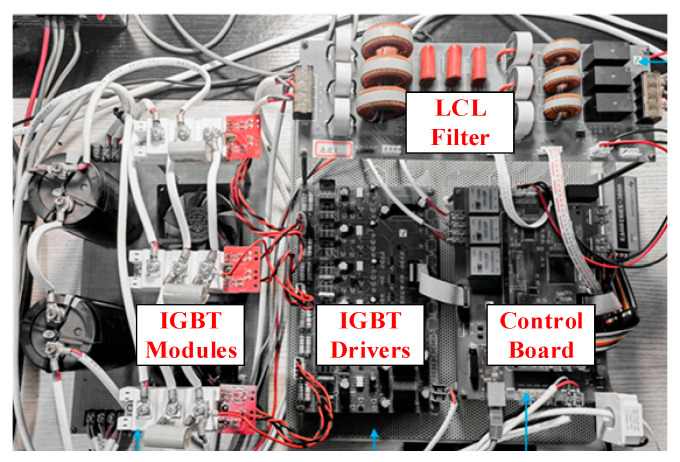
Experimental setup photograph.

To further validate the effectiveness of the proposed method, we construct a 6 kVA three-phase *LCL* grid-connected inverter experimental platform. Figure 18 illustrates the experimental setup. The main circuit topology of the experimental platform is shown in Figure 1, and the parameters are consistent with Table 1. The digital signal processor adopts TI’s TMS320F28335 chip, and the power switch utilizes Infineon’s FF50R12RT4. A programmable AC power supply TPV7006S is connected in series with an inductor to simulate the actual power grid. LEM’s current sensor, LA-55P, is used to sample the grid current, and the grid voltage is sampled through LEM’s voltage sensor LV-25P. The controller adopts a DSP+CPLD structure.

**Figure 19:**
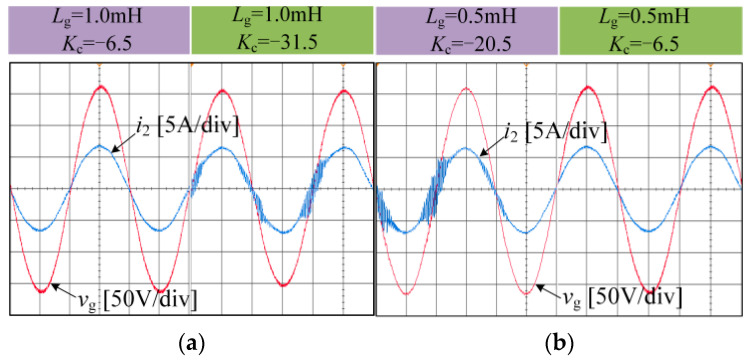
Experimental results of a grid-connected inverter system under different grid impedance and *K*_C_. (**a**) Point A: *L*_g_ = 1 mH, *K*_C_ = −6.5. Point B: *L*_g_ = 1 mH, *K*_C_ = −31.5. (**b**) Point C: *L*_g_ = 0.5 mH, *K*_C_ = −6.5. Point D: *L*_g_ = 0.5 mH, *K*_C_ = −20.5.

The parameters of the current and damping controllers under experimental conditions remain consistent with those of simulation for the four cases of Points A, B, C, and D used for the experiment. Figure 19 illustrates the experimental waveforms of the grid current under four different conditions, and the results closely match the simulation outcomes. The results show that when the grid impedance changes and the system resonance frequency is near the Nyquist frequency, the strategy and parameter design method proposed in this paper can effectively suppress resonance. Compared with the previous related studies, this method not only uses fewer current sensors but also can obtain a larger positive damping region.

**Figure 20:**
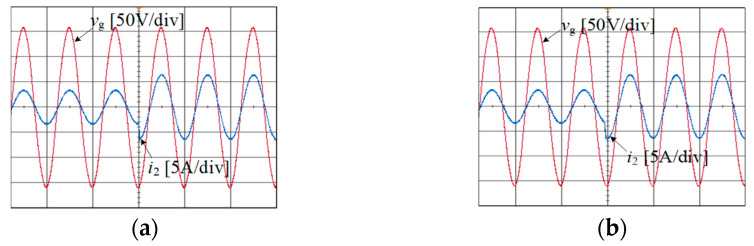
Instantaneous experimental waveform of reference current variation at (**a**) *L*_g_ = 0 mH and (**b**) *L*_g_ = 2 mH.

Figure 20 displays the instantaneous experimental waveforms when the reference current varies with different values of *L*_g_. The instantaneous overshoot and adjustment time of the waveform are small, indicating that the proposed joint damping method exhibits excellent dynamic performance.

## 5. Conclusions

This study focuses on the analysis and research of a joint approach that combines NFGCF active damping and PCC voltage unit feedforward. The theoretical analysis reveals that the proposed method can generate a DDR that spans the entire frequency range, and its upper frequency boundary even exceeds the Nyquist frequency *f*_s_/2. However, the DDR is influenced by variations in grid impedance, potentially causing the system’s resonance frequency to surpass the DDR and leading to non-minimum phase behavior. The research illustrates that the DDR can be adjusted by appropriately designing damping loop control parameters, ensuring the control of non-minimum phase behavior when grid impedance undergoes significant changes. The simulation and experimental results validate the robustness of the joint active damping against impedance changes in the weak grid, enabling the system to maintain stable operation while producing high-quality current waveforms. In this paper, the system control frequency is 10 KHz. Future research will focus whether the method proposed in this article can be effective at higher system frequencies.

## Figures and Tables

**Table 1:** Main parameters of the grid-connected inverter.

Parameter	Value	Parameter	Value
Grid voltage *V*_g_	110 V	Inverter-side inductor *L*_1_	0.6 mH
Input voltage *V*_in_	400 V	Grid-side inductor *L*_2_	0.3 mH
Switching frequency *f*_sw_	10 kHz	Filter capacitor *C*	10 μF
Sampling frequency *f*_s_	10 kHz	*LCL* resonance frequency *f*_r_	3.6 kHz

## Data Availability

The original contributions presented in the study are included in the article, further inquiries can be directed to the corresponding author/s.
